# Impacts of multiple environmental factors on soil bacterial community assembly in heavy metal polluted paddy fields

**DOI:** 10.1038/s41598-024-65678-x

**Published:** 2024-06-26

**Authors:** Mengmeng Zou, Qi Zhang, Fengchun Li, Long Chen, Yifei Qiu, Qiqi Yin, Shenglu Zhou

**Affiliations:** 1 School of Geography and Ocean Science, Nanjing University, 163 Xianlin Road, Nanjing, 210023 Jiangsu People’s Republic of China; 2 Key Laboratory of Coastal Zone Exploitation and Protection, Ministry of Natural Resources, Nanjing, 210024 People’s Republic of China; 3 Testing Center of Shandong Bureau of China Metallurgy and Geology, Jinan, 250014 People’s Republic of China

**Keywords:** Paddy fields, Heavy metals, Cd, Bacterial community, Ecology, Environmental sciences

## Abstract

Soil microorganisms play pivotal roles in driving essential biogeochemical processes in terrestrial ecosystems, and they are sensitive to heavy metal pollution. However, our understanding of multiple environmental factors interaction in heavy metal polluted paddy fields to shape microbial community assembly remain limited. In the current study, we used 16S rRNA amplicon sequencing to characterize the microbial community composition in paddy soils collected from a typical industry town in Taihu region, eastern China. The results revealed that Cd and Pb were the major pollutant, and Proteobacteria, Acidobacteria and Chloroflexi were the dominate indigenous bacterial phyla. Linear regression and random forest analysis demonstrated that soil pH was the most important predictor of bacterial diversity. Mantel analysis showed that bacterial community structure was mainly driven by pH, CEC, silt, sand, AK, total Cd and DTPA-Cd. The constructed bacterial co-occurrence network, utilizing a random matrix theory-based approach, exhibited non-random with scale-free and modularity features. The major modules within the networks also showed significant correlations with soil pH. Overall, our study indicated that soil physiochemical properties made predominant contribution to bacterial community diversity, structure and their association in Cd/Pb polluted paddy fields. These findings expand our knowledge of the key environmental drivers and co-occurrence patterns of bacterial community in polluted paddy fields.

## Introduction

Soils represent one of the largest biodiversity reservoirs on our planet, with each gram of soil harboring a multitude of distinct microbial taxa^1^. These microorganisms form extremely complex and diverse communities that greatly contribute to driving essential ecosystem processes, including maintenance of soil fertility, cycling of nitrogen and carbon, and availability of plant nutrients^2^. However, the global environmental changes exacerbated by intensive human activities have exerted detrimental effects on microbial diversity, consequently weakening ecosystem sustainability and multifunctionality, which are crucial for human well-being^3,4^. Heavy metals possess toxic nature and persistence attributes, and became a significant global environmental concern. Since the onset of the industrial revolution, pollution from anthropogenic heavy metals has dramatically escalated worldwide, resulting in long-term adverse consequences for humans^5,6^. Microorganisms, characterized by their high surface-to-volume ratio, are well-recognized to be sensitive and responsive to heavy metals^7,8^. When concentrations of heavy metals exceed certain thresholds, they can adversely impact microbial growth, morphology, and essential metabolic processes^9^. Consequently, heavy metals act as significant environmental stressors for terrestrial microbial communities.

Recent advancements in molecular technologies have greatly improved our ability to characterize a real microbial community, and gain a deeper understanding of their connections to specific ecological processes^10,11^. Researchers have utilized high-throughput sequencing to evaluate heavy metal induced evolutionary shifts of indigenous microbial community living in metals mining/smelters sites^12–14^, industrial areas^15^, and e-waste sites^16^.With long-term heavy metal exposure, sensitive species suffer from greater pressure and exhibit substantial reductions in abundance and diversity, while certain species may better adapt or even be flourishing by evolved defense mechanisms, ultimately affecting microbial community and the functional traits^17,18^. Exploiting the metabolic processes mediated by these resistant microorganisms holds promise as an alternative approach to address the challenges associated with heavy metal toxicity^19,20^. However, the microbial response to heavy metal stress is not always evident, attributed to the modification of confounding factors present in field scenarios^21,22^. Soil physicochemical properties, for example, can dramatically impact indigenous microorganisms directly or indirectly by controlling heavy metal behavior. Therefore, the combination between soil physicochemical properties and heavy metal stress adds complexity to the understanding of microbial assemble in polluted soils.

Microorganisms inhabiting natural environments do not live as isolated populations, and they associate with each other through various ecological processes, as a whole to carry out many ecosystem functions^10^. In recent decades, the use of microbial ecological networks has become increasingly prevalent in uncovering associations among members of microbial communities, which cannot be fully captured through traditional alpha- and beta-diversity analysis^23,24^. The constructed microbial network consists of two parts: nodes, which represent distinct microbial species, and edges, which exhibited strong positive or negative relationships between nodes. Microbial co-occurrence patterns are embedded in the topological features that reflect the connectivity among microorganisms. Microbial community complexity and stability can be assessed by topological parameters in relation with interested environmental variables^25,26^. Microbial keystone species are considered as occupying crucial roles in maintaining the structure and functioning of microbial communities. The network analysis facilitates their identification in the diverse and largely uncultured microorganisms within microbial communities^27^.

Rice fields, as the largest human-made ecosystems on our planet, encompass approximately 9% of global arable land and feed over 50% of the world's population^28^. Rice cultivation management of periodic flooding and drainage creates alternating oxidized and reduced conditions in paddy fields, which recruit a wide variety of microorganisms^29–31^. Consequently, soil health and agricultural productivity of paddy rice systems are largely determined by these microbial communities^32^. Moreover, paddy soils have also been a significant sink for heavy metals, released by wastewater irrigation, sludge amendment, phosphate fertilizers, mining, and atmospheric deposition^33^. Nevertheless, there still remain a knowledge gap in bacterial community assemblage and co-occurrence patterns in polluted paddy fields. Published studies where heavy metal pollution have exacerbated changes in microbial communities mainly focused on mining sites or e-waste sites^12–14,16^. However, compared with these sites, heavy metals in most polluted paddy fields were not in very high concentrations. Moreover, the key factors that influenced microbial community in polluted soils were different. In this study, soil samples were collected from Dingshu, a typical rural industrial town in Taihu region, eastern China. The extensive use of wastewater irrigation and intensive industrial activities have continuously introduced heavy metals into the cultivated soils in the town. Therefore, the results of our study can enhance the understanding of microbial community in metal-polluted paddy fields. This study aims to the following objectives: (1) characterizing the structure and diversity of bacterial community in polluted paddy soils; (2) determining the association of bacterial community with heavy metals and soil physicochemical properties; (3) unravelling the bacterial co-occurrence networks and identifying the influencing factors. Through this investigation, valuable insights can be gained into heavy metal bioremediation strategies and agricultural management practices in paddy ecosystems.

## Materials and methods

### Study area

Sampling sites were distributed in Dingshu, a county situated on the western shore of Taihu Lake, in southern Jiangsu Province, China. With annual average precipitation of 1177–1500 mm and annual average temperature of 15.7–16.0 °C, this region experiences the East Asian monsoon climate. The predominant soil type in the eastern and central areas of Dingshu is paddy soil, with rice cultivation being managed through a combination of mechanical and minor manual practices. The industrial development in this region is primarily dominated by electromechanical, metallurgical, and ceramic enterprises.

### Sample collection

**Figure 1:**
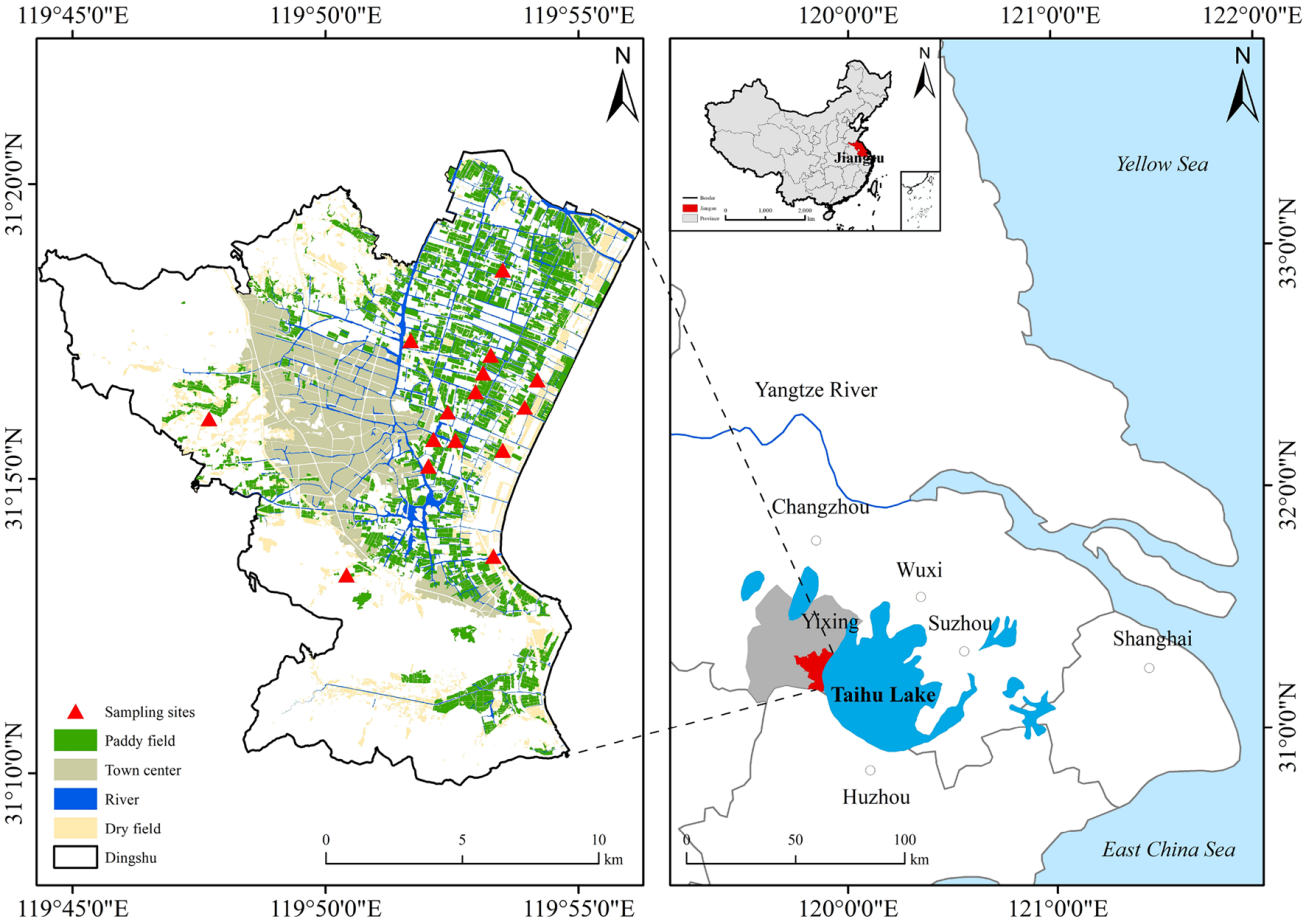
Distribution of sampling sites in the town of Dingshu and map of the study area. This picture was generated in ArcGIS 10.3 (arcgis.com/index.html).

We have established 15 sampling sites, with each site further selecting three sampling fields (Fig. 1 and Table S1). Utilizing a five-point sampling method, a total of 45 paddy soils were collected at the rice harvest time. Specifically, soil samples were collected at the center of the selected paddy field (the midpoint of the diagonal of the field), and at each of the four corners (equidistant from the center on the diagonal). Subsequently, these five collected soil samples were mixed together in the same weight, and raw weight of each soil sample was more than 1 kg. Soil samples for microbial analysis were placed in sterile centrifuge tubes, kept in the insulation box filled with dry ice, and stored at − 80 °C until DNA extraction after immediate transport to the laboratory. The soil samples for heavy metal determination and physiochemical properties were air-dried at room temperature after removing visible gravel and crop roots. Subsequently, the dried soil samples were pounded by wooden sticks, grinded with a ceramic mortar, and then sieved with 10 mesh and 100 mesh nylon sieves. Finally, sieved soil samples were stored into polyethylene zip-lock bags for subsequent chemical analysis.

### Soil physicochemical properties and heavy metals analysis

The soil pH and electrical conductivity (EC) were determined in a soil–water suspension (soil:water ratio = 1:2.5 and 1:5). The size of soil particles was measured using the hydrometer method (HJ 1068-2019, MARA), while soil organic matter (SOM) was determined using the K_2_Cr_2_O_7_ oxidation method (NY/T 1121.6-2006, MOAC). Soil cation exchange capacity (CEC) were assessed using the ammonium acetate (NH_4_OAc) method (GB 15618-2018). Total nitrogen (TN) and available nitrogen (AN) were examined with modified Kjeldahl method and alkali diffusion method, respectively (LY/T 1228-2015). Available phosphorus (AP) was analyzed by molybdenum antimony colorimetry (NY/T 1121.7-2014). Available potassium (AK) was extracted with NH4OAc, and subsequently detected using a flame photometer (NY/T 889-2004).

To determine the metal concentrations, the prepared soil specimens underwent digestion using a combination of HCl, HNO_3_, HF, and HClO_4_. Extractable heavy metals were obtained using diethylene triamine pentaacetate (DTPA). The soil levels of Cu, Cd, Zn, and Pb, both in their total and DTPA-extractable fractions, were quantified by inductively coupled plasma mass spectrometry (ICP-MS; Thermo Fisher Scientific, USA). To ensure quality assurance, duplicate samples, reagent blanks, and utilized reference materials (GBW 07406a and GBW 07405) were applied.

### DNA extraction, amplification, and sequencing

According to the kit’s protocol, HiPure Soil DNA Kits were used to extract the microbial DNA (Magen, Guangdong, China). The concentration and purity of extracted DNA was assessed with a NanoDrop spectrophotometer (ND-2000, Thermo Fisher Scientific, USA).

The 16S rRNA variable regions were amplified by PCR using the following primer pairs: 341F (CCTACGGGNGGCWGCAG) and 806R (GGACTACHVGGGTATCTAAT) for 5 min at 95 °C, 30 cycles of 1 min at 95 °C, 1 min at 60 °C, and 1 min at 72 °C, and 7 min at 72 °C. Following a set methodology, the purified amplicons were mixed in equimolar quantities and put through paired-end sequencing (PE250) on an Illumina platform. The bacterial raw sequences were available in the National Center for Biotechnology Information (NCBI) Sequence Read Archive (accession number PRJNA1091049). After reads filtering, reads assembly and raw tag filtering, obtained effective tags were searched against the reference database to perform reference-based chimera checking using UCHIME algorithm, and all chimeric tags were removed. Using the UPARSE process (v9.2.64), the effective tags produced from the sequencing data were grouped into operational taxonomic units (OTUs) based on a similarity criterion of 97%. The UCHIME technique was used to remove any chimeric tags in order to guarantee data integrity. The remaining tags, also known as effective tags, were then chosen for additional examination. The tag sequence with the highest abundance within each cluster was chosen as the representative sequence. The RDP classifier (v2.2) was used in conjunction with a naïve Bayesian model to categorize the representative OTU sequences into the appropriate species. The SILVA database (v138.1) was used in the categorization procedure.

### Microbial ecological networks construction

Network construction was performed using the online integrated Network Analysis Pipeline (iNAP)^34^ (Galaxy (denglab.org.cn). To enhance the accuracy of predictions and reduce spurious results, the inclusion of OTUs in the network were limited to those present in at least 90% of the samples. Pearson correlation was performed for calculation of the association of pairwise OTUs and the adjacent correlation matrix was constructed. Threshold of the correlation matrix was automatically determined using a random matrix theory (RMT)-based method to avoid subjectivity of determination, and the selected cutoff vulue in our study was 0.77 (Fig. S1). Subsequently, network matrix and associated edges attributes were obtained, using the cutoff value. Topological features of microbial ecological network were characterized by a range of parameters, including nodes, links between nodes, average path distance (GD), average clustering coefficient (avgCC), average degree (avgK), modularity and ect. All nodes and links in the constructed network are rewired 100 times to generate the random networks, then selected topological properties of random networks and empirical networks were compared. Module division was carried out with the provided method (greedy modularity optimization) in iNAP pineline. Finally, the microbial network was visualized using Gephi software (v 0.10), facilitating a visual representation of the relationships between OTUs. Additionally, the within-module connectivity (Z) and among-module connectivity (P) for each node were calculated for classifing its topological roles within the whole network.

### Statistical analysis

To evaluate the heavy metal pollution level in single and multiple metals, respectively, the contamination factor (*CF*) and pollution load index (*PLI*) were used^35^. According to Eq. (1), *CF* is calculated by comparing the determined concentration of a particular metal in sampling paddy fields to its background value in the study region. According to Eq. (2), *PLI* value is calculated by taking the square root of selected heavy metal *CF* values. Pollution occurrence is often indicated by a *PLI* value of > 1.0.

$$CF = \frac{Heavy\;metal\;in\;sampling\;soil}{{Heavy\;metal\;in\;background\;soil}}$$

$$PLI = \left( {CF_{1} \times CF_{2} \times \cdots \times CF_{n} } \right)^{1/n}$$

The calculation of microbial α-diversity indices including Sobs, Chao1, ACE, and Shannon was done using QIIME (v 1.9.1). The β-diversity of microorganisms was quantified using the Bray–Curtis dissimilarity index. Using linear regressions, the relationships between the measured soil characteristics and the microbial diversity indices were examined. Random forest (RF), which can evaluate the importance of soil variables in predicting the microbial diversity indices, was carried out using the R package “randomForest” (R version 4.3.2, https://www.r-project.org/). Variation partitioning analysis (VPA), which was utilized to ascertain the impact of key environmental conditions on microbial community structure, was aided by the R package “vegan”. Mantel tests were used to determine the significance of Spearman's rank correlations between microbial populations and environmental parameters using the R package “vegan”. The relationships between examined soil variables and the dominant bacterial phyla were displayed with Spearman correlation heatmap, and visualized the R package “ggplot2”.

## Results

### Soil physiochemical properties and heavy metal concentrations

Table 1 listed the physiochemical properties of collected soil samples. Soil pH values exhibited a range from acid to slightly acid, with a mean of 5.43. pH values of most samples were below 6.0 (except YX_2, pH: 6.35). Although acid soil is conducive for rice growth and many rice plant species grow well at the pH of 5.5, low pH can increase heavy metal bioavailability. Most soil samples were rich in organic matter (> 30 g/kg), and the mean value (36.53 g/kg) was slightly higher than that of China (32.4 g/kg). Moreover, CEC ranged from 2.52 to 16.17 cmol/kg, and the mean value (8.78 cmol/kg) was below 10 cmol/kg. EC is widely used indicator for assessing soil salinity levels. In the samples collected, the EC values ranged from 65.84 to 271.57 ms/m, indicating the presence of varying degrees of salinity in the soil. All soil samples were classified as clay loam soil or clay soil, according to the international standard for soil texture classification. TN, AN, AP and AK contents were, on average, 1.96 g/kg, 140.37 mg/kg, 95.53 g/kg and 90.66 g/kg, respectively, placing them within the range of the high or middle class. The wide range observed in most of the examined soil variables could be attributed to the diverse farming practices employed by small-holder farmers.

**Table 1:** Soil physicochemical properties in sampling fields.

Samples	SOM (g/kg)	pH	CEC	EC (ms/m)	Clay (%)	Silt (%)	Sand (%)	TN (g/kg)	AN (mg/kg)	AP (mg/kg)	AK (mg/kg)
YX_1	30.22	4.97	6.17	111.41	19.45	31.30	49.25	1.84	112.22	68.50	75.67
YX_2	38.59	6.35	9.63	178.14	23.39	33.15	43.46	2.45	133.15	108.12	122.94
YX_3	52.32	5.75	14.83	132.06	28.88	36.18	34.94	2.87	189.53	196.85	117.32
YX_4	50.70	5.53	13.56	131.50	27.55	35.55	36.90	2.88	188.97	192.40	121.28
YX_5	27.00	5.46	6.54	81.57	20.17	31.55	48.28	1.63	132.52	71.47	76.47
YX_6	30.27	5.41	7.64	204.07	20.92	32.23	46.85	1.90	114.69	72.45	72.45
YX_7	33.60	5.23	8.79	114.13	22.39	32.94	44.66	2.01	127.69	86.38	78.46
YX_8	32.23	5.51	7.20	114.90	21.12	31.93	46.95	1.10	125.08	44.55	88.93
YX_9	40.92	5.25	9.26	165.93	23.49	33.21	43.30	1.03	170.29	58.41	94.49
YX_10	31.36	4.82	5.92	149.31	20.50	31.13	48.38	1.09	118.86	41.45	71.02
YX_11	29.87	5.41	9.22	140.46	22.46	33.20	44.34	1.06	121.43	43.49	95.69
YX_12	42.30	5.40	12.93	116.94	26.38	35.24	38.39	2.63	142.09	131.56	100.69
YX_13	31.18	5.54	8.57	86.24	23.39	32.73	43.88	2.20	135.26	97.23	82.82
YX_14	35.99	5.20	4.58	97.87	20.64	30.17	49.19	1.98	127.05	75.93	78.34
YX_15	41.42	5.59	6.92	85.62	21.22	31.79	46.99	2.68	166.65	144.18	83.39

The total concentrations ranged from 0.22 to 9.80 mg/kg for Cd, 6.98–18.86 mg/kg for Cu, 28.62–61.76 mg/kg for Pb and 33.59–83.62 mg/kg for Zn, respectively (Table 2). According to GB 15618-2018, Cd concentrations in most samples exceeded the risk screening value, except for YX_8, YX_10 and YX_13, which were 0.4 mg/kg for 5.5 < pH ≤ 6.5, and 0.3 mg/kg for pH ≤ 5.5, respectively. In addition, total Pb and Cd concentrations in all soil samples were higher than the soil background values of Taihu region (Pb = 20.78 mg/kg, Cd = 0.11 mg/kg), and the soil samples demonstrated the moderate concentrations of Zn, but relatively lower concentrations of Cu. In the present study, all investigated paddy fields had *PLI* above 1 (Table 2), implying the appearance of metal pollution. The highest PLI value was recorded in YX_4 (2.77). The average *CFs* of Cu, Cd, Zn and Pb were 0.57, 6.00, 0.84 and 1.92, respectively. Thus, the sampling paddy fields were polluted with Cd and Pb. Based on our previous research, it was determined that atmospheric deposition resulting from industrial activities and transportation emerged as the primary sources of Cd and Pb in the agricultural soils of this region^36^. Soil samples with high total concentrations of heavy metals also had high levels of DTPA-extractable metals. DTPA-extractable concentrations of Cd, Cu, Pb and Zn were in the range of 0.09–6.03 mg/kg, 3.00–9.40 mg/kg, 3.47–14.72 mg/kg and 1.05–4.66 mg/kg (Table 2).

**Table 2:** Heavy metal concentrations in sampling fields (mg/kg).

Samples	T-Cd	A-Cd	T-Cu	A-Cu	T-Pb	A-Pb	T-Zn	A-Zn	*PLI*
YX_1	1.62	0.95	8.53	4.64	35.81	6.13	33.59	2.39	1.51
YX_2	2.21	1.20	11.34	4.41	35.39	5.53	81.63	1.83	2.11
YX_3	0.46	0.19	25.37	9.40	61.76	9.09	82.58	4.07	2.07
YX_4	9.80	6.03	10.58	5.86	55.11	9.57	48.10	3.24	2.77
YX_5	0.78	0.34	8.41	3.09	28.62	3.47	35.76	2.67	1.21
YX_6	0.55	0.24	6.98	3.47	39.00	4.65	45.02	2.16	1.18
YX_7	4.01	1.38	13.59	3.57	42.94	4.06	67.27	2.01	2.64
YX_8	0.29	0.12	13.68	3.00	33.15	4.12	57.65	2.24	1.24
YX_9	0.30	0.11	10.39	4.85	38.17	4.69	46.16	2.23	1.13
YX_10	0.22	0.08	13.83	4.32	32.01	6.44	59.24	2.79	1.17
YX_11	0.31	0.11	14.72	4.96	39.78	5.98	59.24	1.71	1.32
YX_12	0.35	0.09	14.50	4.65	47.20	5.96	53.31	1.05	1.40
YX_13	0.23	0.09	14.74	6.69	33.76	5.98	70.01	2.97	1.23
YX_14	0.45	0.20	18.86	4.83	54.59	14.72	83.62	4.66	1.86
YX_15	0.37	0.18	13.19	4.58	37.66	8.84	63.63	2.66	1.37

T-Cd, T-Cu, T-Pb and T-Zn represent the concentrations of total Cd, Cu, Pb and Zn, respectively; A-Cd, A-Cu, A-Pb and A-Zn represent the concentrations of DTPA-extractable Cd, Cu, Pb and Zn, respectively.

### Diversity and structure of bacterial community in paddy fields

The valid tags of all samples varied in the range of 110,842–127,886, with a mean of 118,396. OTUs ranged from 3531 to 5903, with a mean of 4841. Robust representation of diverse groups with a minimum coverage exceeding 0.980, affirming the exceptional accuracy and close proximity to the actual values in the sequencing and comparison outcomes (Table S2). Furthermore, Sobs, Shannon, Chao1, and ACE (α-diversity indexes) varied in the ranges of 3531–5903, 9.082–10.512, 4284–6955, and 4310–7039, respectively (Table S2). Variation coefficients were 9.04%, 2.89%, 8.76% and 9.25% Shannon, Sobs, Chao and ACE, respectively, implying a low variability in the evenness, richness, and diversity of bacterial community in paddy fields.

**Figure 2:**
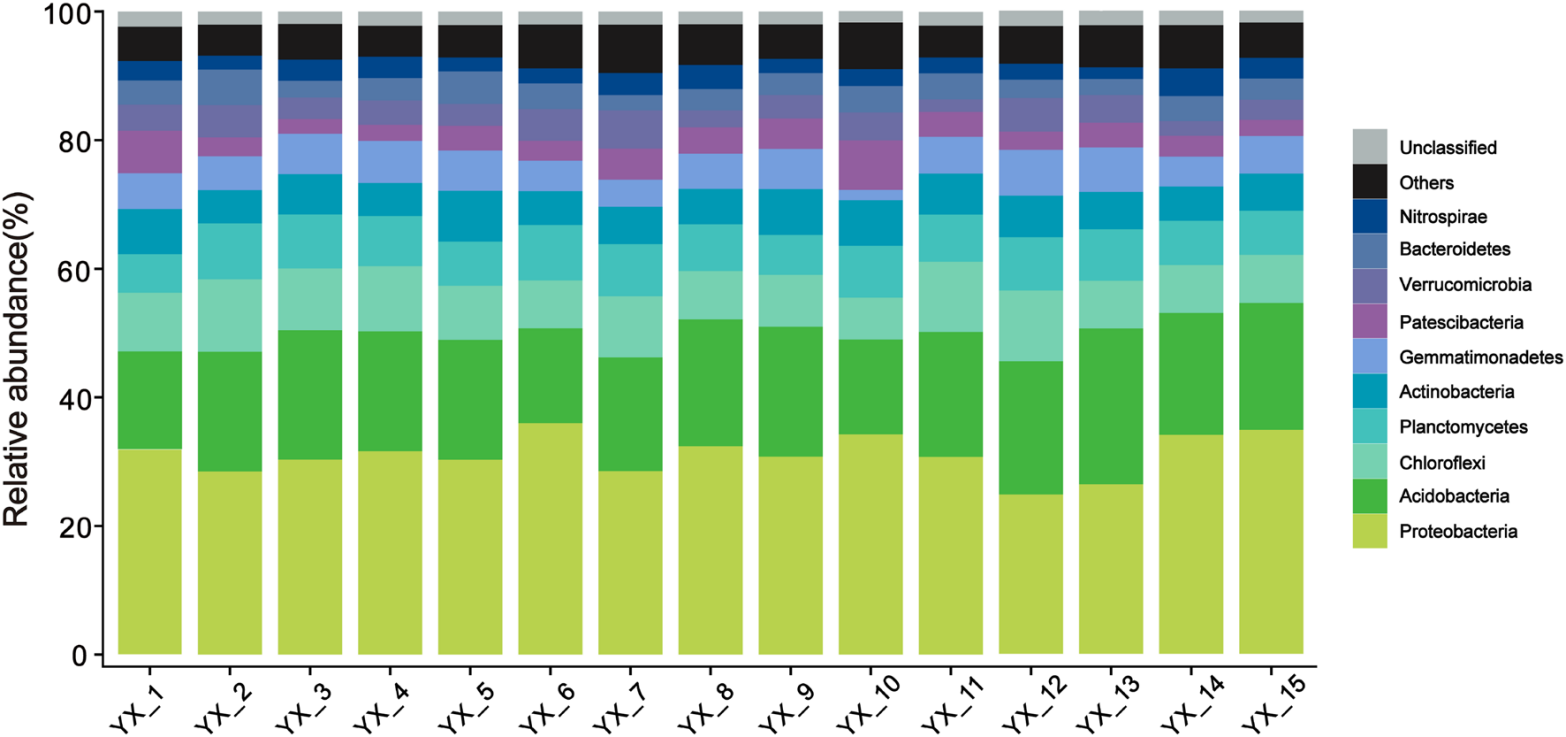
The taxonomic composition of bacterial community at the phylum level.

Taxonomic classification of all identified OTUs revealed the presence of 48 phyla, with bacteria (99.4%) overwhelmingly dominating the 16S rRNA gene sequences. Archaea accounted for a small proportion (0.6%) of the assigned sequences. Among the detected OTUs, a significant majority (90%) belonged to the following 10 dominant phyla: Proteobacteria (24.82–36.0%), Acidobacteria (14.70–24.14%), Chloroflexi (6.53–11.29%), Planctomycetes (5.98–8.66%), Actinobacteria (5.17–7.95%), Gemmatimonadetes (1.60–7.12%), Patescibacteria (2.27–7.68%), Verrucomicrobia (1.94–5.91), Bacteroidetes (2.44–5.59%), and Nitrospirae (1.80–4.30%) (Fig. 2). Among all the samples, Proteobacteria occupied the highest proportion, followed by Acidobacteria, and both of them accounted for about 50% of total sequence data. The variability degree of dominant phyla was moderate, with an average variation coefficient of 28%.

### Examined soil variables influencing bacterial diversity and community structure

**Figure 3:**
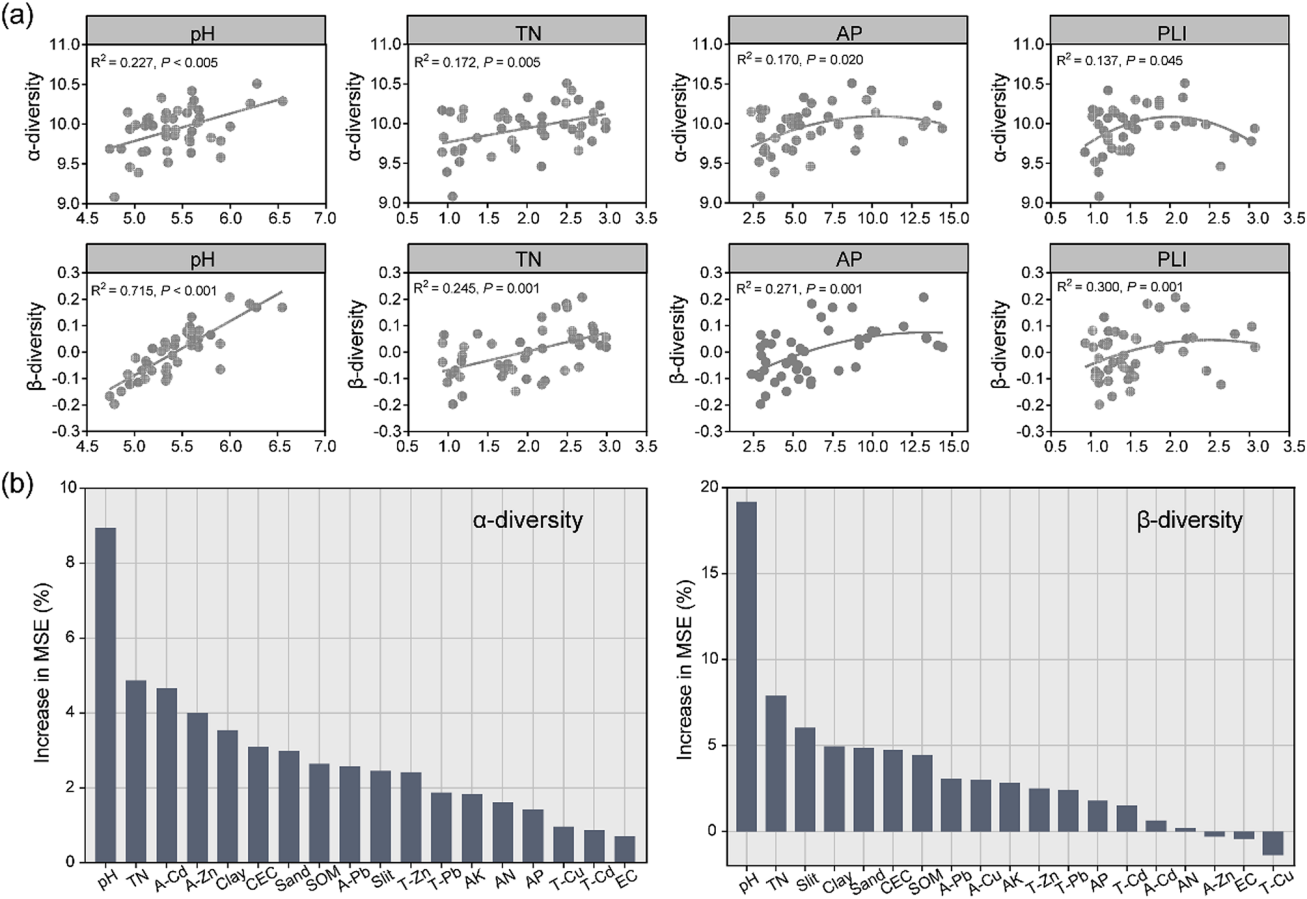
Effect of heavy metals and soil properties on bacterial community diversity; (**a**) linear regressions between bacterial α-diversity (Shannon), β-diversity (NMDS1) and pH, TN, AP, PLI. (**b**) RF analysis to evaluate the relative importance of examined soil variables to predict bacterial community diversity. Percentage increase in the MSE (mean squared error) of variables was used to estimate the importance of these predictors, and higher MSE% values implied more important predictors.

Bacterial α-diversity and β-diversity indexes were both significantly correlated with soil variables (measured by the pH, TN, and AP) and PLI. Furthermore, β-diversity displayed significant relations with other soil properties, including SOM, CEC, soil texture (clay, silt and sand), AN, AP and AK (Table S3). Bacterial α-diversity exhibited positive linear correlations with soil pH and TN, respectively (pH: R^2^ = 0.227, *P* < 0.005; TN: R^2^ = 0.172, *P* = 0.005) (Fig. 3a). Similarly, bacterial β-diversity also demonstrated positive correlations with soil pH and TN (pH: R^2^ = 0.715, *P* < 0.001; TN: R^2^ = 0.245, *P* = 0.001). Bacterial diversity and soil AP concentrations had a quadratic association (R^2^ = 0.170, *P* = 0.020), and the maximum value was observed at approximately 10.0 mg/kg of AP. In addition, bacterial α-diversity showed an increasing trend with increasing PLI up to around PLI 2.0, beyond which there was no further increase in α-diversity despite higher soil PLI levels. Lower AP levels (below approximately 12.5 mg/kg) and lower PLI levels (below 2.5) appeared to promote bacterial β-diversity. Notably, soil pH demonstrated stronger impacts on both bacterial α-diversity and β-diversity in paddy fields, as evidenced by higher R^2^ values compared to TN, AP, and PLI (Fig. 3b).

**Figure 4:**
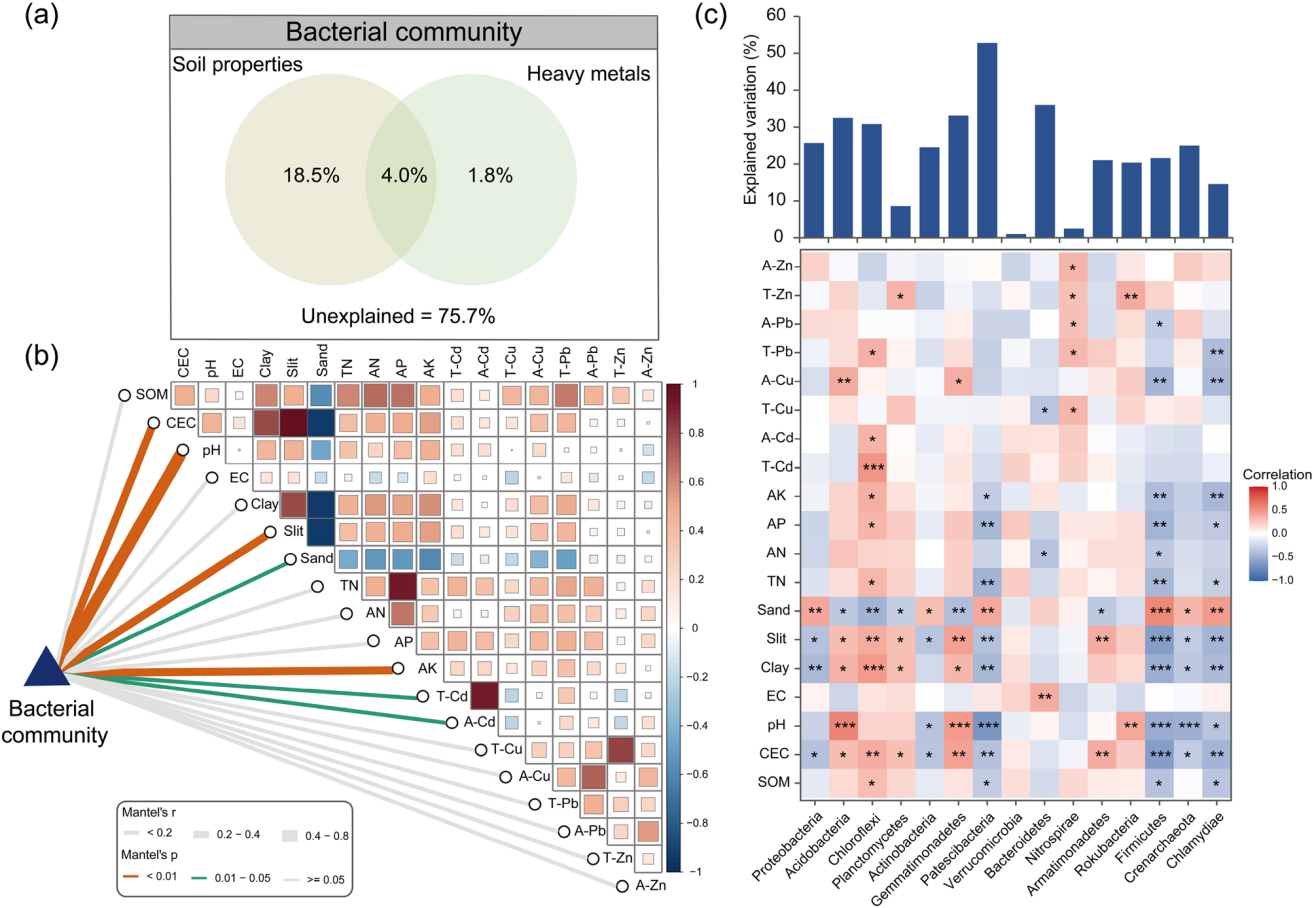
Relationships between bacterial community structure and examined soil variables. (**a**) VPA analysis to evaluate the variation of bacterial community structure explained by soil properties and heavy metals. (**b**) Partial Mantel tests showed association between bacterial community structure and soil variables. (**c**) Heatmap of Spearman’s correlations coefficients between soil variables and dominant bacterial phyla.

According to the result of VPA analysis, soil properties, heavy metals and their interaction accounted for 18.5%, 1.8% and 4.0% of the variation in bacterial community compositions, respectively (Fig. 4a). Soil properties possessed a higher contribution to the bacterial community compared to heavy metals. However, it is worthy to mention that a significant portion of the variation (75.7%) remained unexplained. Additionally, the partial Mantel test (Fig. 4b) identified notable factors associated with the microbial community. The results confirmed that soil pH, CEC, silt, sand, AK, T-Cd and A-Cd were key factors shaping bacterial community in paddy soils, and soil pH was the most relevant factor of examined soil variables (r = 0.687, *P* < 0.001).

The relative abundance of dominant bacterial phyla exhibited different degrees of correlations with examined soil variables (Fig. 4c). Soil pH, CEC, clay, silt and sand were significantly correlated with dominant bacterial phyla, including Acidobacteria, Gemmatimonadetes, Patescibacteria, Firmicutes, Crenarchaeota and Chlamydiae. Patescibacteria, Firmicutes and Chlamydiae were negatively correlated with soil TN, AP and AK. Chloroflexi exhibited a positive correlation with Cd in both the total and DTPA-extractable fractions. Cd is recognized as the major pollutant present in the sampling fields. Nitrospirae showed positive correlations with T-Cu, T-Pb, A-Pb, T-Zn and A-Zn. FS-MRM indicated that the variations in the relative abundance of dominant bacterial phyla could be partly explained by examined soil variables. Notably, the explained variation ranged greatly from 1.00% (Verrucomicrobia) to 52.78% (Patescibacteria).

### Bacterial co-occurrence network and its association with soil environmental factors

**Figure 5:**
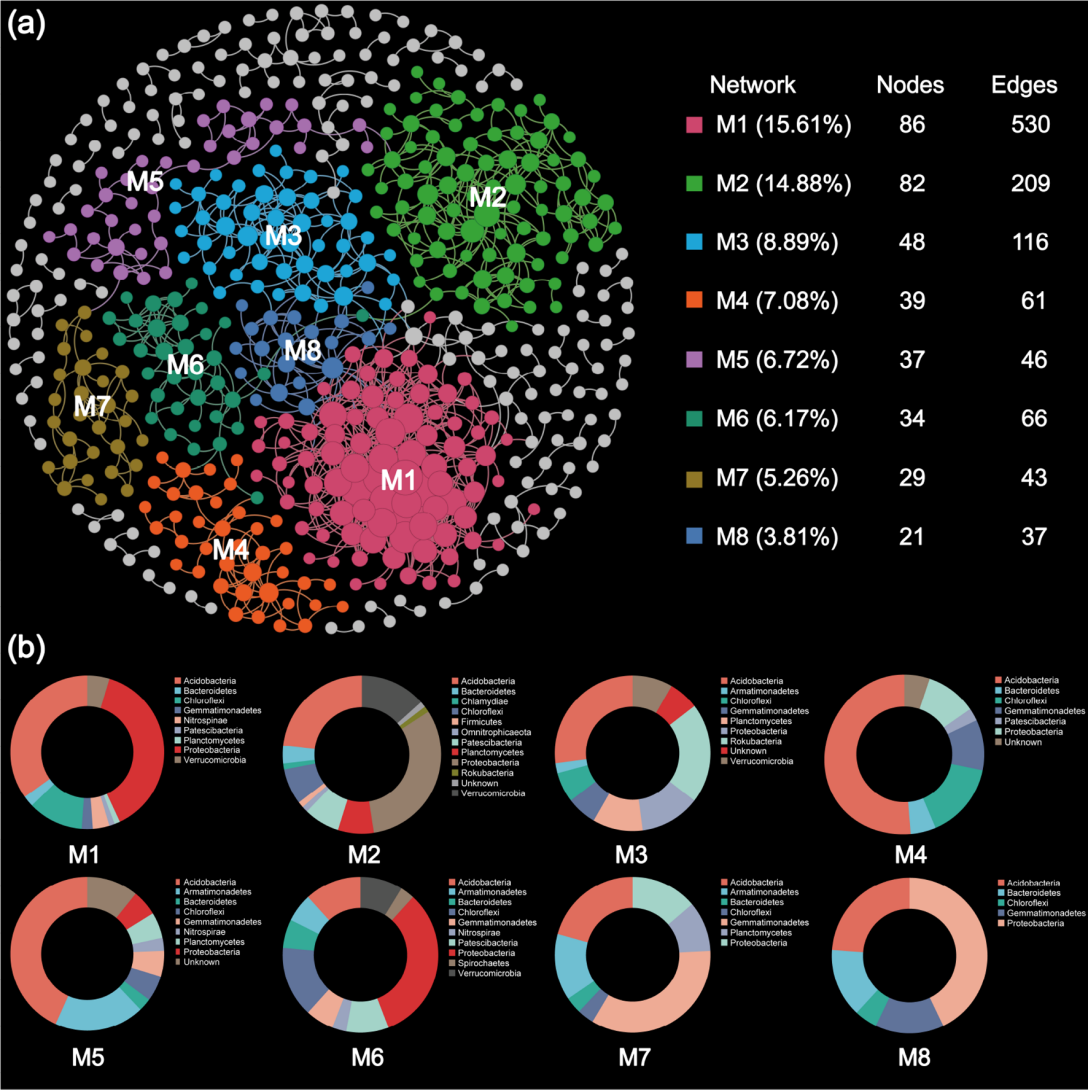
The bacterial co-occurrence network constructed by core OTUs. (**a**) Eight dominant modules (M1–M8) were exhibited in different colors. (**b**) Taxonomic profiles of eight dominant modules.

A bacterial co-occurrence network was constructed, comprising of 551 OTUs and 1252 edges. The connectivity of constructed network fitted the pow law model (R^2^ = 0.979), implying scale-free attribute of the network. Furthermore, selected topological properties of constructed network were significantly higher than those of random networks, and this confirmed the non-randomness of network structure (Table 3). By considering the proportion of phyla that constitute more than 5% of the total nodes, it was observed that six phyla, namely, Proteobactria, Acidobacteria, Chloroflexi, Gemmatimonadetes, Verrucomicrobia and Actinobacteria, collectively accounted for ~ 75% of the nodes in the constructed networks. In addition, this network displayed a great degree of modularity, with 68.2% of the nodes assigned to only 8 out of the total 62 modules (Fig. 5a). Each module was characterized by distinct taxonomic profiles as OTUs were annotated to their representative microbial taxa. These 8 modules were composed of 15 different phyla, highlighting the diverse taxonomic composition within the network. Among these phyla, Proteobacteria, Acidobacteria, Chloroflexi, Gemmatimonadetes and Verrucomicrobia were dominant (Fig. 5b). Acidobacteria, Proteobacteria, and Chloroflexi were detected in each of all 8 modules, indicating they had a high co-occurrence incidence. Three phyla such as Chlamydiae, Firmicutes and Omnitrophicaeota were only detected in Module 2, while Spirochaetes only existed in Module 6.

**Table 3:** Topological properties of the ecological network of bacterial community and their associated random networks.

Empirical network								Random networks		
St	Nodes	Link	R^2^ of Power-law	avgK	GD	avgCC	Modularity	GD ± SD	avgCC ± SD	Modularity ± SD
0.77	551	1282	0.979	4.653	8.537	0.293	0.739	3.809 ± 0.042	0.001 ± 0.002	0.442 ± 0.005

*avgK* average connectivity, *avgCC* average clustering coefficient, *GD* average geodesic distance, *St* threshold value.

**Figure 6:**
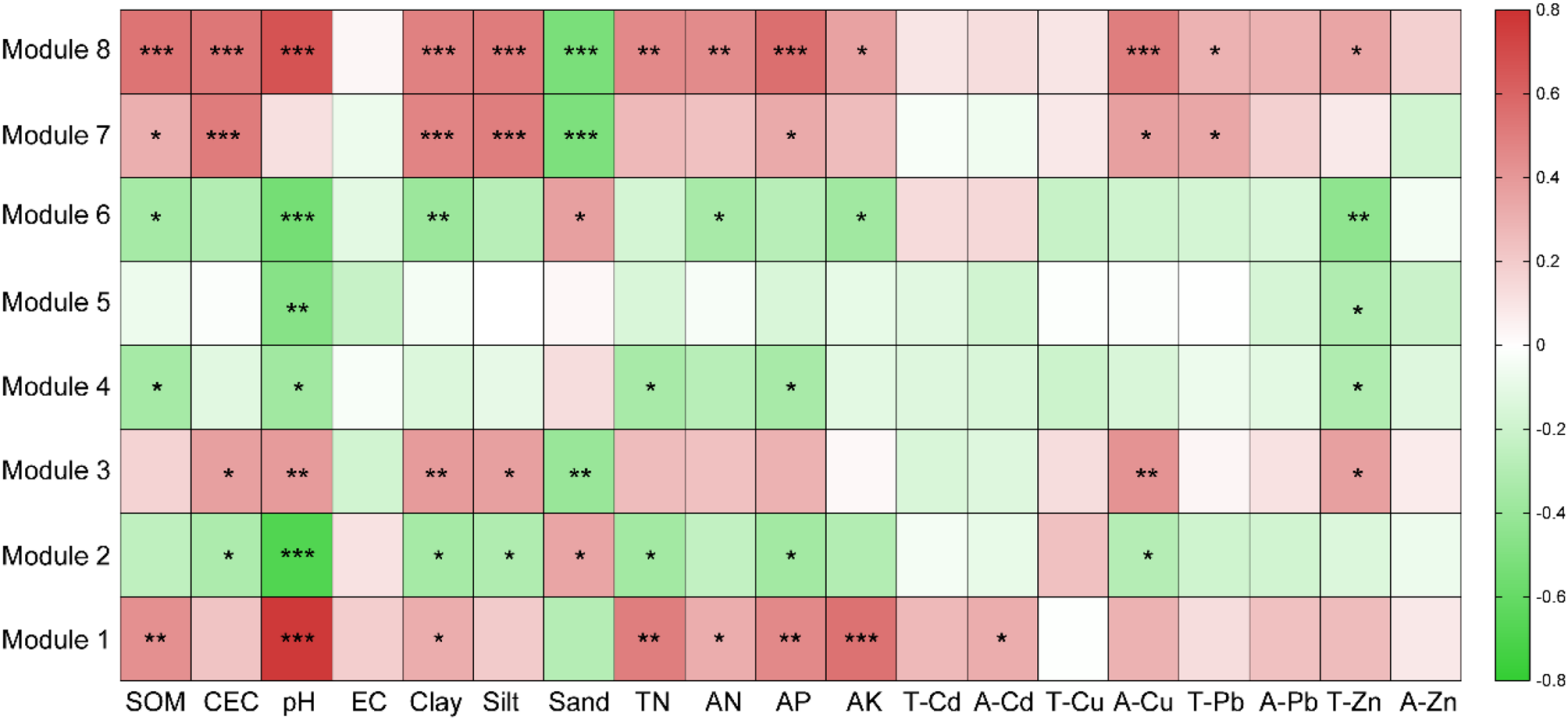
The association of eight dominant modules with soil variables and revealed by Mantel test.

Mantel test was performed to identify the association of network interactions in major modules with soil variables and. The results indicated that inter-interactions within the modules, excluding module 7, exhibited significant correlations with soil pH (Fig. 6). Module 1 and module 8 were both sensitive to soil nutrients K, N, and P. Soil texture were closely related to the interactions of OTU in modules. A-Cd significantly affected the interactions of OTU in module 1. A-Cu and T-Zn exerted remarkable impacts on inter interactions in most modules.

Based on the Zi and Pi values of nodes within constructed network, topological roles played by all nodes were divided into four types: modules hubs, connectors, network hubs, and peripherals (Fig. S2). Over 98% of all nodes were identified as peripherals, this result implied most nodes only occupying a few links. Seven module hubs, which connect other nodes within a specific module, were assigned to Acidobacteria, Proteobacteria, Armatimonadetes, Chloroflexi, Actinobacteria and Gemmatimonadetes (Table 4). Network connectors link different modules and bring together many different microbial niches. There were two connectors belonging to Proteobacteria and Chloroflexi, respectively. Notably, no network hub OTUs were identified in this study. Furthermore, module hubs (OUT_397, OUT_803) and connectors (OUT_485, OUT_530) had low relative abundances (< 0.1%), implying that rare species living in the bacterial community may also hold significant importance in ecological functions.

**Table 4:** Microbial taxonomic information of module hubs and connectors in the co-occurrence work and their abundance.

ID	Generalists	Phylum	Class	Order	family	Genus	Tags abundance (%)
OTU_11	Module hubs	Acidobacteria	Acidobacteriia	Acidobacteriales	*Koribacteraceae*	*Candidatus_Koribacter*	0.639
OTU_40	Module hubs	Proteobacteria	Gammaproteobacteria	Betaproteobacteriales	*SC-I-84*	*Unclassified*	0.300
OTU_108	Module hubs	Armatimonadetes	Chthonomonadetes	Chthonomonadales	*Unclassified*	*Unclassified*	0.151
OTU_111	Module hubs	Chloroflexi	Ktedonobacteria	Ktedonobacterales	*JG30-KF-AS9*	*Unclassified*	0.148
OTU_155	Module hubs	Actinobacteria	Actinobacteria	Unclassified	*Unclassified*	*Unclassified*	0.115
OTU_397	Module hubs	Proteobacteria	Deltaproteobacteria	Sva0485	*Unclassified*	*Unclassified*	0.047
OTU_803	Module hubs	Gemmatimonadetes	S0134_terrestrial_group	Unclassified	*Unclassified*	*Unclassified*	0.023
OTU_485	Connectors	Chloroflexi	Ktedonobacteria	C0119	*Unclassified*	*Unclassified*	0.037
OTU_530	Connectors	Proteobacteria	Gemmatimonadetes	Gemmatimonadales	*Gemmatimonadaceae*	*Unclassified*	0.035

## Discussion

### Effects of heavy metal pollution on soil bacterial community and diversity

It was not surprising that Cd, one of the most well-known hazardous heavy metals, significantly affected the composition of bacterial community in selected paddy fields. Although Cd has not been proven to be essential for microbial metabolic processes, it can impose detrimental effects even at low concentrations by damaging microbial DNA, proteins, and enzymatic activities^37,38^. Microorganisms can evolve various mechanisms to defense unfavorable heavy metal exposure. Exclusion through permeability barriers, intra- and extracellular sequestration, active transport efflux pumps, and a decrease in the sensitivity of cellular targets to Cd ions are the main mechanisms by which Cd resistance develops^39–42^. The dominant phylum Chloroflexi exhibited positive correlations with total Cd and DTPA-Cd (Fig. 4c). Furthermore, at the genus level, *Ellin6067*, *Candidatus_Koribacter*, *Bryobacter*, *Occallatibacter*, *Anaerolinea* and *Baciius* also showed significant correlations with DTPA-Cd and total Cd (Table S4). With the exception of *Anaerolinea*, which is the dominant genus within the phylum Chloroflexi, the associations of these genera with soil Cd were all negative, suggesting the toxicity of Cd to most bacteria. *Anaerolineaceae*, due to their ability to interact *syntrophically* with methane metabolism microbiota, could play a crucial role in available Cd precipitation^43,44^. Toxicity of heavy metals in soil are commonly governed by their bioavailability, and while available Pb concentrations were relatively low, it was not extraordinarily toxic to microorganisms^45^. Despite being one of the major pollutants in selected paddy fields, the obvious impacts of Pb on the bacterial community were not detected.

In field environments, multiple heavy metals generally coexist in paddy soils. The relationships between PLI and bacterial α- and β- diversity indexes can be described by quadratic models. The response of microorganisms to heavy metals is partly influenced by their concentrations^46,47^. Zn and Cu are confirmed as essential metals for microbial growth and metabolism, especially at low concentrations. The relative abundance of dominant phyla, including Planctomycetes, Nitrospirae, and Rokubacteria exhibited positive correlations with the total concentration of Zn. Similarly, a positive relationship between Nitrospirae and total Cu was also observed (Fig. 4c). Zhao et al.^48^ demonstrated that relationships between microbial community diversity and multiple heavy metal pollution were not simply negative, and the highest diversity indexes were recorded in soils with moderate levels of mixed pollution. Lin et al.^49^ observed that paddy soils with moderated and severe metal-polluted levels had higher bacterial α-diversity indexes than lightly polluted paddy fields.

### Effects of soil properties on soil bacterial community and diversity

Our study detected the impacts of soil pH on bacterial community structure and diversity in sampling paddy fields were predominant, even far more than that of Cd. The primary control of soil pH in indigenous microorganisms were emphasized in multiple scenarios, such as e-waste sites^16^, metal-polluted sites^49^, and arable lands^50^. Relationships between microbial diversity and soil pH can fit a quadratic model, and neutral soil pH tends to support higher diversity^51–53^. However, the pH values of sampling paddy fields ranged relatively narrowly from 4.97 to 6.35, and we observed positive correlations between bacterial diversity indexes (Shannon and NMDS1) and soil pH. The intracellular pH of many microorganisms is nearly neutral, additionally, the ranges of optimal pH conditions for microbial survival and function are generally narrow^54^. Deviation from this optimum pH range can impose a range of physiological constraints on microorganisms, limiting the growth of non-survivable taxa outside their preferred pH range or leading to changes in competitive outcomes^55^. The different response patterns of dominant bacteria to pH observed in Fig. 4c. may support the above analysis. In addition, soil pH may serve as an integrative factor, reflecting the overall soil conditions rather than directly influencing bacterial structure. In our study, soil pH significantly correlated with CEC (r = 0.477, *P* < 0.01), clay (r = 0.459, *P* < 0.01), silt (r = 0.477, *P* < 0.01), sand (r =  − 0.469, *P* < 0.01), TN (r = 0.377, *P* < 0.05), AP (r = 0.404, *P* < 0.01), and AK (r = 0.484, *P* < 0.01). These soil factors clearly made contribution to shape bacterial community assembly (Figs. 3, 4).

The results of linear regression and Mantel test analysis demonstrated that soil nutrients (TN, AP, and AK) also influenced the diversity and composition of bacterial communities, as consistent with previous research findings. Nitrogen and phosphorus elements are necessary for microbial biomolecules synthesis (e.g., DNA, RNA and ATP)^56^, while potassium serves as an intracellular activator in microorganisms^57^. The availability of these nutrients can lead to shifts in the abundance of copiotrophic-oligotrophic taxa^58,59^. Additionally, significant correlations between soil texture (silt and sand), CEC and bacterial community were observed through the Mantel test. Soil texture has long been recognized as a key factor influencing various soil processes, including C sequestration and storage, nutrient retention, water infiltration, and structural development^60^. In the soil system, microorganisms predominantly inhabit the surfaces and voids of soil particles, which are composed of varying sizes. According to Xia et al.^61^, the second most significant factor in determining the composition of the soil microbial community, after soil pH, is soil texture. Microbial diversity was higher in finer textured soils than in coarser textured soils because of the higher nutrient content and diverse microenvironments in the small particle fraction, which provide effective protection for soil microorganisms^62–64^. Jiang et al.^16^ found that soil pH, clay percentage and CEC explained 59.3% of the variance of soil bacterial community diversity at the e-waste site, which was seriously contaminated by heavy metals. In our studies, bacterial α-diversity (Shannon) displayed insignificant relation with soil clay, silt and sand. The less impact of texture on bacterial diversity may be due to the strong influence of pH on the diversity^51^. We recorded significant associations between clay, silt, sand and 15 dominant phyla, with 9, 10, and 10 phyla, respectively, exhibiting significant associations with these soil texture components. And different microbes show preference for different particle size bins (Fig. 4c).

### Co-occurrence pattern of bacterial community

The phyla Proteobacteria, Chloroflexi, and Acidobacteria dominated the bacterial taxonomic composition examined in all samples. This pattern was also observed in other metal-polluted places, as these phyla possess either inherent or acquired resistance to heavy metals^65–67^. The majority of heavy metal resistance genes were detected in Proteobacteria, one of the most diverse group and largest in the bacteria domain. The metal tolerance of Proteobacteria is achieved through various mechanisms, such as the exportation of metals via ion channels, pumps, transporters, or reduction through redox reactions^68,69^. Proteobacteria are copiotrophs, and they can efficiently utilize carbon derived from plants. With increased organic matter in soil, members of Proteobacteria tend to have a higher relative abundance^70^. A rise in the abundance of Proteobacteria usually indicates a positive effect on microbial resistance to toxic substances and soil health. Members of the chloroflexi phyla are extensively dispersed in a variety of habitats and are crucial to biogeochemical cycles involving multiple elements, including sulfur, nitrogen, and carbon^71,72^. The positive association of Chloroflexi populations with heavy metals have been documented in other studies^13,15^. Acidobacteria populations can survive in harsh environments and are considered oligotrophs. They exhibit metabolic versatility, including the ability to biodegrade plant residues, participate in the iron cycle, possess photosynthetic capacity, and engage in monocarbon metabolism^73^. Negative correlations between Acidobacteria and soil pH were recorded in previous studies^74,75^. However, our study observed opposite results, possibly attributed to different responses of Acidobacteria subgroups to soil pH^54,73^. Regarding the dominant genus within Acidobacteria, different relationships with pH were observed. *Candidatus_Koribacter* showed a positive correlation (r = 0.486, *P* = 0.001), while *Bryobacter* (r =  − 0.377, *P* = 0.011), *RB41* (r =  − 0.736, *P* < 0.001), and *Occallatibacter* (r =  − 0.429, *P* = 0.003) exhibited negative correlations. These results indicated a predominance of negative correlations between the dominant Acidobacteria genus and pH.

The non-random of constructed microbial network implied that microorganisms harboring in the sampled paddy fields tend to exhibit correlations more frequently than would be expected by chance alone, possibly attributable to the influence of deterministic processes in microbial community assembly^23^. In addition, our network exhibited topological features of scale-free, small-world, and modularity. These properties have significant implications for the stability and resilience of ecosystems^76^. These general network features have been observed in microbial ecological networks across various spatial scales in numerous studies^77–79^. More than half of the nodes in the entire network were associated with Proteobacteria (25.8%), Acidobacteria (18.9%), and Chloroflexi (10.5%). The high incidence of co-occurrence among these phyla further emphasizes their importance in maintaining the structure and function of microbial communities.

In microbial ecology, the term “module” refers to a grouping or cluster of microbial species that are tightly interconnected within themselves but have looser connections with nodes in other modules. The ecological mechanisms regulating microbial communities, such as habitat selection and niche filtration, are explained by this compartmentalization^78,80^. By examining the association of microbial modules with interested environmental variables, we can better comprehend the effect of abiotic factors on microbial interactions, revealing crucial aspects of the overall microbial community that may not be immediately apparent^81^. We identified eight major modules, of which seven were found to be relevant to soil pH. The strong correlations of soil pH with major network modules were in line with previous where highlighted the effect of soil pH on the relative abundance of modules, modules and topological feature of microbial co-occurrence networks^82–84^. Our results supported that soil pH shaped microbial community at the level of microbial occurrence networks. Notably, Module 1, which showed a positive association with soil DTPA-Cd, was predominantly composed of Proteobacteria, Acidobacteria and Chloroflexi. This finding suggests that these microorganisms possess the ability to interact with each other, potentially contributing to their resistance against Cd toxicity. Furthermore, we discovered positive correlations between soil nutrient levels (e.g., AK, AN, TN, AP and SOM) and Module 1 and Module 8 (Fig. 5). This indicates that the members of these two modules might occupy niches enriched with these nutrients^85^. Interestingly, these modules exhibited contrasting responses to examined soil variables, which could be attributed to subgroup-specific responses. Therefore, acquiring more detailed taxonomic information is necessary to gain a deeper understanding of these patterns.

Connectors and Module hubs function as mediators, regulators, or adaptors in the microbial networks, serving as bridges within their own modules or between different modules. Although we did not detect a network hub, which aligns with other network research^79,86^, we identified seven module hubs and two connectors as keystone taxa. Nevertheless, a majority of these module hubs or connectors remained unclassified even at the genus level, making it challenging to confidently infer their potential functions. Interestingly, keystone taxa often exhibit relative low abundance, and a study conducted by Shi et al.^87^ also observed the pattern. Rare or less abundant species can play critical roles in shaping functional diversity, genetic diversity, and ecosystem stability in the face of environmental disturbances^88^. Hence, solely focusing on abundant taxa would disregard the importance of these less common but significant species. Manipulating the keystone species identified through microbial networks holds promise as a viable approach for managing agriculture in metal-polluted paddy ecosystems.

## Conclusion

In the current study, we investigated the impacts of multiple environmental factors on bacterial community assembly in Cd/Pb polluted paddy fields from an industrial town. Proteobacteria, Acidobacteria and Chloroflexi dominated the indigenous bacterial composition. Soil physiochemical properties prevail over heavy metals to shape bacterial community structure and diversity, especially soil pH emerging as the most influential factor. Non-random with scale-free and modularity features occurred in constructed ecological network, and significant correlations were detected between major modules and soil pH, providing a new insight into bacterial associations in heavy metal polluted paddy fields. These findings can enhance our understanding of the mechanism to drive microbial community assembly in heavy metal polluted paddy fields.

### Supplementary Information

Supplementary Information.

## Data Availability

Sequence data that support the findings of this study have been deposited in the National Center for Biotechnology Information (NCBI) Sequence Read Archive (accession number PRJNA1091049).
