# Supplementary material for: Impacts of multiple environmental factors on soil bacterial community assembly in heavy metal polluted paddy fields
Source: Sci Rep. 2024 Jun 26;14:14696. doi: 10.1038/s41598-024-65678-x (PMC11208537; doi:10.1038/s41598-024-65678-x)
Supplement: Supplementary file 1 — Supplementary Information. [file 41598_2024_65678_MOESM1_ESM.docx]

**Supplementary materials**

**Impacts of multiple environmental factors on soil bacterial community assembly in heavy metal polluted paddy fields**

Mengmeng Zou^a,b^, Qi Zhang^a, b^, Fengchun Li^c^, Long Chen^a, b^, Yifei Qiu^a,b^, Qiqi Yin^a,b^, Shenglu Zhou^a,b^*

*^a^ School of Geography and Ocean Science, Nanjing University, Nanjing 210023, China*

*^b^ Key Laboratory of Coastal Zone Exploitation and Protection, Ministry of Natural Resources, Nanjing 210024, China*

*^c^ Testing Center of Shandong Bureau of China Metallurgy and Geology, Jinan 250014, China*

***Corresponding author**

Name: Shenglu Zhou; E-mail: [zhousl@nju.edu.cn](mailto:zhousl@nju.edu.cn)

Postal address: School of Geography and Ocean Science, Nanjing University, 163 Xianlin Road, Nanjing, Jiangsu 210023, P.R. China

**Contents**

**Table S1 Geographic location of sampling sites**

**Table S2 Bacterial diversity indexes in paddy fields**

**Table S3 Spearman’s correlation coefficients between soil physicochemical properties or PLI and bacterial diversity indexes**

**Fig. S1 Selected cut-off value for bacterial network**

**Table S4 Spearman’s correlation coefficients between relative abundance of dominant bacterial genera and soil Cd concentrations**

**Fig. S2 *Z*-*P* plot exhibiting the distribution of OTUs based on their topological roles. Each point represents an OTU**

**Table S1**

**Geographic location of sampling sites**

| Soil samples | Coordinates |
| --- | --- |
| YX-1 | N 119°54′11″, E 31°16′41″ |
| YX-2 | N 119°52′34″, E 31°15′39″ |
| YX-3 | N 119°52′2″, E 31°15′13″ |
| YX-4 | N 119°52′8″, E 31°15′40″ |
| YX-5 | N 119°53′30″, E 31°15′29″ |
| YX-6 | N 119°53′56″, E 31°16′13″ |
| YX-7 | N 119°52′25″, E 31°16′8″ |
| YX-8 | N 119°53′19″, E31°13′41″ |
| YX-9 | N 119°52′58″, E 31°16′29″ |
| YX-10 | N 119°53′16″, E 31°17′6″ |
| YX-11 | N 119°53′7″, E 31°16′48″ |
| YX-12 | N 119°51′41″, E 31°17′21″ |
| YX-13 | N 119°53′30″, E 31°18′33″ |
| YX-14 | N 119°50′25″, E 31°13′22″ |
| YX-15 | N 119°47′42″, E 31°16′1″ |

**Table S2**

**Bacterial diversity indexes in paddy fields**

| **Samples** | **Sobs** | **Shannon** | **Chao** | **Ace** | **Good_coverage** |
| --- | --- | --- | --- | --- | --- |
| **YX-1** | 4645 | 9.869 | 5637 | 5643 | 0.986 |
| **YX-2** | 5203 | 10.355 | 6059 | 6090 | 0.986 |
| **YX-3** | 5013 | 10.072 | 6257 | 6395 | 0.980 |
| **YX-4** | 4820 | 9.915 | 5883 | 5944 | 0.985 |
| **YX-5** | 5005 | 9.932 | 6105 | 6302 | 0.981 |
| **YX-6** | 4899 | 9.952 | 5767 | 5828 | 0.986 |
| **YX-7** | 4546 | 9.756 | 5482 | 5568 | 0.986 |
| **YX-8** | 5186 | 10.139 | 6267 | 6391 | 0.981 |
| **YX-9** | 4297 | 9.563 | 5273 | 5312 | 0.987 |
| **YX-10** | 4351 | 9.640 | 5211 | 5237 | 0.988 |
| **YX-11** | 4607 | 9.664 | 5525 | 5605 | 0.987 |
| **YX-12** | 4512 | 9.816 | 5396 | 5500 | 0.986 |
| **YX-13** | 4783 | 9.949 | 5729 | 5789 | 0.985 |
| **YX-14** | 5285 | 10.121 | 6418 | 6527 | 0.983 |
| **YX-15** | 5476 | 10.288 | 6627 | 6788 | 0.981 |

**Table S3**

**Spearman’s correlation coefficients between soil physicochemical properties or PLI and bacterial diversity indexes**

|  | **SOM** | **CEC** | **pH** | **EC** | **Clay** | **Silt** | **Sand** | **TN** | **AN** | **AP** | **AK** | **PLI** |
| --- | --- | --- | --- | --- | --- | --- | --- | --- | --- | --- | --- | --- |
| **α-diversity (Shannon)** | 0.134 | 0.061 | **0.476^**^** | -0.135 | 0.048 | 0.049 | -0.051 | **0.415^**^** | 0.141 | **0.337^*^** | 0.024 | **0.326^*^** |
| **β-diversity (NMDS1)** | **0.425^**^** | **0.478^**^** | **0.845^***^** | 0.029 | **0.513^***^** | **0.455^**^** | **-0.513^***^** | **0.495^**^** | **0.334^*^** | **0.497^**^** | **0.455^**^** | **0.538^***^** |


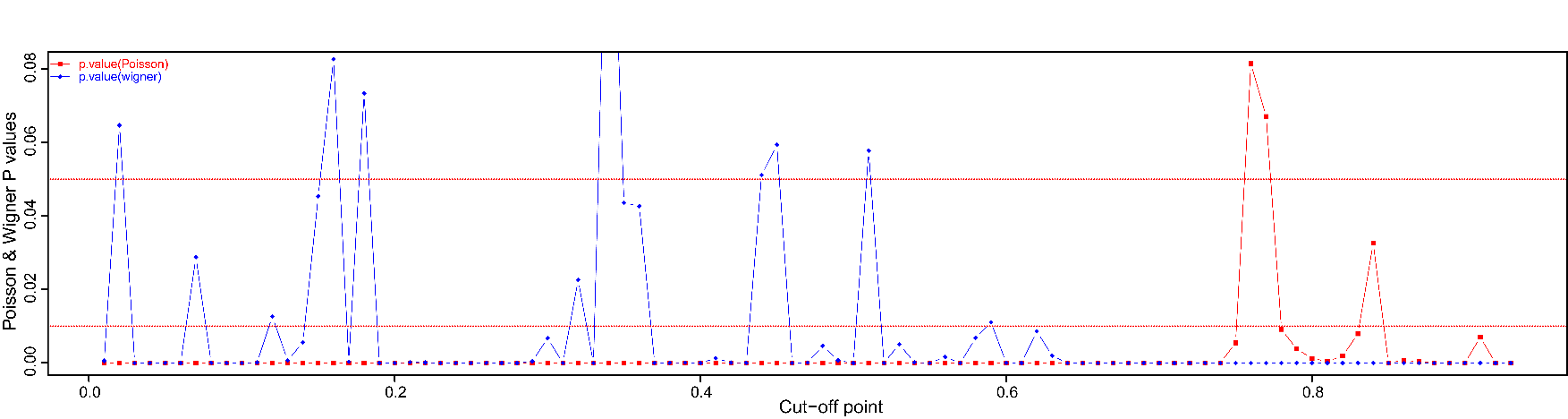


**Fig. S1 Selected cut-off value for bacterial network**


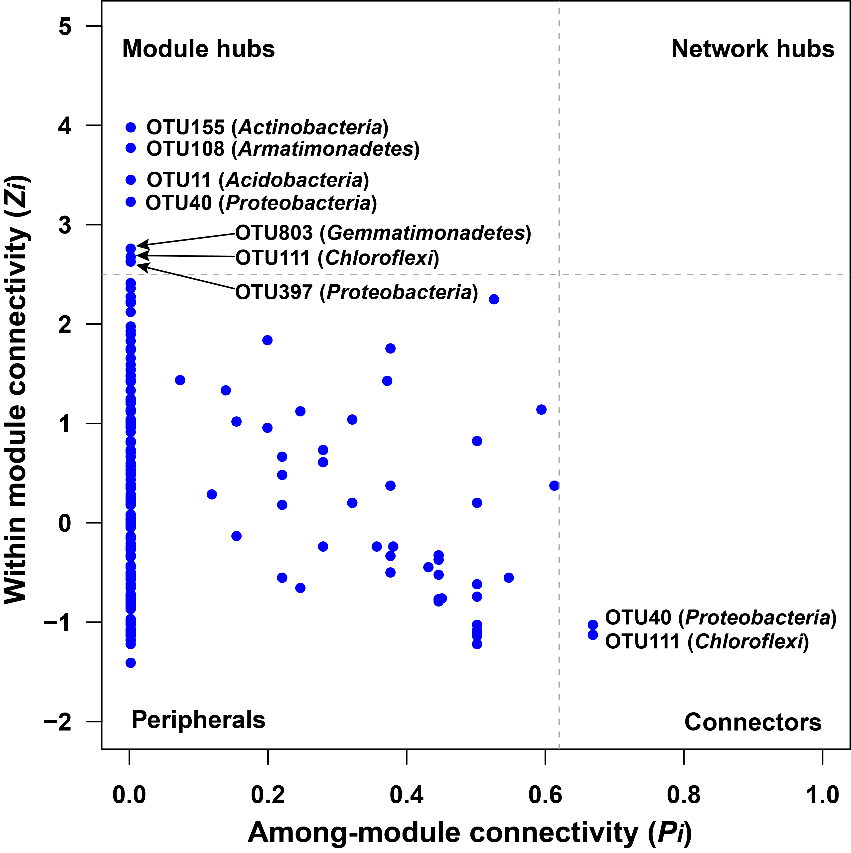


**Fig. S2 *Z*-*P* plot exhibiting the distribution of OTUs based on their topological roles. Each point represents an OUT.** **Nodes attributes could be divided into four types: module hubs (Zi > 2.5), network hubs (Zi > 2.5 and Pi > 0.62), connectors (Pi > 0.62) and peripherals (Zi<2.5 and Pi<0.62).**

**Table S4**

**Spearman’s correlation coefficients between relative abundance of dominant bacterial genera and soil Cd concentrations**

| **Phylum** | **Genus** | **T-Cd** | | **A-Cd** | |
| --- | --- | --- | --- | --- | --- |
|  |  | **r** | ***p*** | **r** | ***p*** |
| *p_Proteobacteria* | *g_Ellin6067* | **-0.395**** | 0.009 | **-0.420**** | 0.005 |
| *p_Acidobacteria* | *g_Candidatus_Koribacter* | **-0.338*** | 0.027 | **-0.370*** | 0.015 |
| *p_Acidobacteria* | *g_Bryobacter* | **-0.301*** | 0.044 | **-0.327*** | 0.028 |
| *p_Acidobacteria* | *g_Occallatibacter* | **-0.327*** | 0.029 | **-0.392**** | 0.008 |
| *p_Chloroflexi* | *g_Anaerolinea* | **0.468**** | 0.001 | **0.330*** | 0.027 |
| *p_Firmicutes* | *g_Bacillus* | **-0.513***** | <0.001 | **-0.459**** | 0.002 |
